# Practical Recommendations for Implementing Remote Patient Monitoring for Patients Receiving Bispecific Antibodies for Multiple Myeloma

**DOI:** 10.2196/95949

**Published:** 2026-11-05

**Authors:** Cindy Varga, Eric Schaefer

**Affiliations:** 1 Department of Hematologic Oncology and Blood Disorders, Atrium Health Levine Cancer Institute, 1021 Morehead Medical Drive, Charlotte, NC, 28204, United States, 1 980-442-4363; 2 Department of Medical Oncology, Highlands Oncology Group, Fayetteville, AR, United States

**Keywords:** remote patient monitoring, bispecific antibodies, multiple myeloma, immunotherapy, outpatient monitoring

## Abstract

Bispecific antibodies are novel immunotherapies approved for the treatment of patients with relapsed or refractory multiple myeloma (MM). While these therapies induce deep and durable responses, they are associated with adverse events, such as cytokine release syndrome and immune effector cell–associated neurotoxicity syndrome, that require specific protocols for monitoring, prevention, and treatment. Due to the potential risk of cytokine release syndrome and immune effector cell–associated neurotoxicity syndrome, current US labeling recommends inpatient administration during step-up dosing of bispecific antibodies, which typically occurs in academic centers and can often restrict the number of patients that can be treated. The administration of bispecific antibodies in the outpatient and community setting can enhance the patient experience and reduce resource use, which is a barrier to the widespread uptake of these treatments. With bispecific antibodies being evaluated in earlier lines of therapy for MM, the broader use of these agents will underscore the need for more accessible and less resource-intensive options that ensure safe outpatient administration. In this Viewpoint, we aim to provide practical recommendations built from our own real-world perspectives and case examples for institutions seeking to implement remote patient monitoring (RPM) strategies to support the outpatient dosing of bispecific antibodies in patients with MM. We discuss how the use of RPM has the potential to improve adverse event detection and management, improve patient outcomes, and decrease resource use and costs. While specific approaches will vary based on the needs of each treatment center, our experiences suggest that RPM implementation can be safe and feasible for both academic and community practices.

## Introduction

Bispecific antibodies (teclistamab, talquetamab, linvoseltamab, and elranatamab) are novel immunotherapy agents approved for the treatment of relapsed or refractory multiple myeloma (MM) [1-8]. In heavily pretreated patients, bispecific antibodies induce deep and durable responses with predictable safety profiles [9]. In addition to common hematologic and nonhematologic adverse events (AEs) typically associated with MM therapies, patients treated with bispecific antibodies are at increased risk of cytokine release syndrome (CRS), immune effector cell–associated neurotoxicity syndrome (ICANS), and infections, all of which require patients to be closely monitored and managed [10-13]. AEs such as neutropenia and hypogammaglobulinemia contribute to the increased infection risk in patients receiving bispecific antibodies [14]. Recommended strategies to mitigate infection risk include vaccinations; the use of antibacterial, antifungal, and antiviral prophylaxis; and intravenous immunoglobulin supplementation regardless of immunoglobulin G blood levels [14,15]. In addition, reducing the frequency of bispecific antibody dosing in responding patients may help reduce the risk of infection [16]. CRS and ICANS events tend to occur during step-up dosing (SUD) of bispecific antibodies or following the first full treatment dose [9]. Therefore, bispecific antibodies are generally administered in hospitals and academic medical centers, with US labeling suggesting hospitalization for 24 to 48 hours following SUD to monitor for signs and symptoms of CRS and ICANS [4-6,8]. Prophylactic tocilizumab administered before the first dose [17,18] and pretreatment with dexamethasone, acetaminophen, and diphenhydramine before each SUD and the first full treatment dose [4-6,8] can help reduce CRS risk.

Inpatient treatment administration and subsequent monitoring for AEs can be resource intensive for hospital teams, limiting the number of patients who can receive treatment with bispecific antibodies at any one time. Academic medical centers may be able to better handle the resource burden and costs of administering and monitoring treatment than community institutions, yet not all patients have access to academic centers [19]. Implementing bispecific antibody treatment in other practice settings will help expand patient access, reduce inpatient hospitalizations, and improve the patient experience [20,21]. These novel immunotherapies are being evaluated in earlier lines of therapy [9], with the National Comprehensive Cancer Network Clinical Practice Guidelines in Oncology updating their recommendations in 2026 to include the use of teclistamab in combination with daratumumab for treatment of MM after one prior line of therapy including lenalidomide and a proteasome inhibitor [22]. Strategies that support the safe and effective administration of bispecific antibodies in outpatient clinics and community institutions are needed to facilitate the widespread use of these treatments in a broader population of patients with MM.

Accumulating evidence shows that bispecific antibodies can be safely administered in the outpatient setting. Outpatient vs inpatient monitoring for tarlatamab, a bispecific antibody approved for the treatment of small cell lung cancer, was directly compared in a phase 1 trial [23]. The safety profile of patients who received tarlatamab in the outpatient setting was similar to that observed with inpatient dosing, with no increase in the risk or severity of CRS or ICANS, and most CRS events were managed without hospitalization or an emergency department visit. In patients with MM, several real-world studies have shown outpatient administration of bispecific antibodies to be feasible, safe, and potentially cost-effective with appropriate prophylaxis and patient management [24-29].

Administration of bispecific antibodies in outpatient and community settings is feasible when supported by an appropriate infrastructure with careful planning, clear protocols, and a well-trained multidisciplinary team [21,30-32]. It is important that patients and their caregivers receive comprehensive support and education, including information on how to recognize the signs and symptoms of CRS and ICANS [21]. A practice-based road map has been developed for the outpatient administration of bispecific antibodies in patients with MM, with recommendations for infrastructure setup and logistics, including bed capacity and resource allocation; multidisciplinary team composition and training; patient selection; patient and caregiver education; SUD procedures; and AE prophylaxis, monitoring, and treatment [21]. The guidelines can be adapted to various health care environments considering available resources, infrastructure, and patient populations for each center.

Remote patient monitoring (RPM) is an example of one strategy being used to implement outpatient dosing programs [30]. RPM uses noninvasive devices (eg, wearables), electronic patient-reported outcomes (ePROs) manually entered into a digital device (eg, tablet or smartphone), and/or invasive devices (eg, cardiac monitors) to obtain patient physiological data and other information about their condition outside the traditional health care environment [33]. The information captured allows the care team to monitor conditions and escalate care as needed if a patient experiences an AE [34]. The use of RPM for patients receiving bispecific antibodies in the outpatient setting has the potential to expand patient access to these treatments, improve AE detection and management, decrease inpatient hospitalization time, streamline clinician workload (eg, by reducing the need for phone check-ins), lower overall health care costs, and improve patient outcomes [35].

Emerging evidence is demonstrating the benefit of RPM in patients with MM. For example, a small academic study evaluated the effectiveness of RPM wearable devices compared with standard-of-care monitoring to detect early CRS in patients with MM receiving bispecific antibodies and found that RPM was more sensitive and led to faster CRS detection than standard-of-care monitoring [36]. A retrospective single-center analysis of patients with MM treated with bispecific antibodies found that patients who received outpatient SUD with prophylactic dexamethasone combined with structured RPM had lower rates of CRS (13% vs 54%), recurrent CRS (0% vs 11%), and ICANS (7% vs 21%) compared with those who received standard inpatient SUD [37]. A larger study of patients with MM treated with teclistamab across 3 Mayo Clinic locations demonstrated the safety and feasibility of outpatient SUD using a technology package for RPM that comprised a tablet and Bluetooth-capable devices (thermometer, blood pressure monitor, and pulse oximeter) [31]. Among the 57 patients who completed outpatient SUD, CRS and ICANS occurred in 31.6% and 3.5%, respectively; all cases resolved, and no patients discontinued teclistamab treatment. In another Mayo Clinic study of 34 patients with MM treated with teclistamab and talquetamab SUD in an outpatient setting, including outpatient tocilizumab to treat CRS, 47% of patients were able to remain as outpatients even with an occurrence of CRS. In an evaluation of resource use, it was estimated that if all doses of teclistamab, talquetamab, and tocilizumab were given on an outpatient basis, it would have resulted in a total net revenue improvement of US $115,004 over the 7-month period analyzed [38].

In this Viewpoint, we aim to provide practical recommendations based on our own experiences for hematology and oncology physicians seeking to implement RPM for patients receiving bispecific antibodies for MM and provide support for the safe and feasible use and adoption of bispecific antibodies in the outpatient and community setting. We provide 2 case studies from our institutions that demonstrate successful RPM adoption at clinical practices using different systems and resourcing models. While these examples are specific to the administration of bispecific antibodies, the RPM approaches described herein can be applied to other therapies that require close monitoring for AEs. Of note, we discuss RPM implementation from a primarily US-based perspective, and therefore, the recommendations may not be broadly applicable to other countries due to differences in health care and payer systems.

## Case Study Examples

The Atrium Health Levine Cancer Institute implemented an outsourcing program with Current Health and their care model Hospital at Home to monitor and provide care to patients in their homes [39]. Patients planned for bispecific antibody SUD received a thermometer, blood pressure cuff, pulse oximeter, and wearable monitor to measure heart and respiratory rate, as well as a tablet to input their vital signs every 4 hours during waking hours. On the days between SUD, a mobile integrated health worker examined patients at home, evaluated the immune effector cell–associated encephalopathy score, and coordinated a virtual visit with a Hospital at Home internist. Patients also had round-the-clock virtual access to a team of Hospital at Home dedicated nurses, who are trained to manage phone calls from patients via a call service. Eight milligrams of prophylactic dexamethasone were administered the day after each SUD, and patients experiencing grade 1 CRS events were managed with acetaminophen and additional doses of dexamethasone as needed. Patients who developed persistent fever despite the above interventions, higher-grade CRS, or any-grade ICANS required hospital evaluation.

In addition to their established RPM program, Atrium Health is also exploring a temperature patch that measures core body temperature continuously for up to 90 days. The patch can be worn 24 hours a day, 7 days a week and is designed to alert the patient via mobile app if a fever develops, with the intent of identifying symptoms faster than self-reporting by the patient. This approach has shown to be an effective method for fever detection in patients with hematologic malignancies. Preliminary data on patients at high risk of febrile neutropenia who had recently undergone chemotherapy and stem cell transplantation show that continuous temperature monitoring with a wearable patch can be used for the accurate and early detection of fevers in an outpatient setting [40]. At Atrium Health, the temperature patch is currently being studied in patients treated with chimeric antigen receptor (CAR) T-cell therapy and may eventually be used to monitor all patients with cancer at risk of fever, including those receiving bispecific antibody treatment.

The Highlands Oncology Group is an independent practice that uses an ePRO approach for RPM in partnership with Canopy Care. Canopy’s RPM system implements a smart device (eg, smartphone or tablet) for eligible patients, or phone calls for patients who do not have a smart device, to report symptoms to their care team. Symptom notifications are monitored by a dedicated triage nursing staff [41]. Patients with symptoms that exceed a predetermined severity threshold undergo further evaluation, including telephone triage, urgent outpatient office evaluation, or referral to the emergency department or hospital. Patients with symptoms of CRS or ICANS are referred to a partner hospital recommended by the practice.

An overview of the overall process for implementing RPM in the outpatient and community setting is illustrated in Figure 1, and specifics are discussed in the sections that follow.

**Figure 1.**
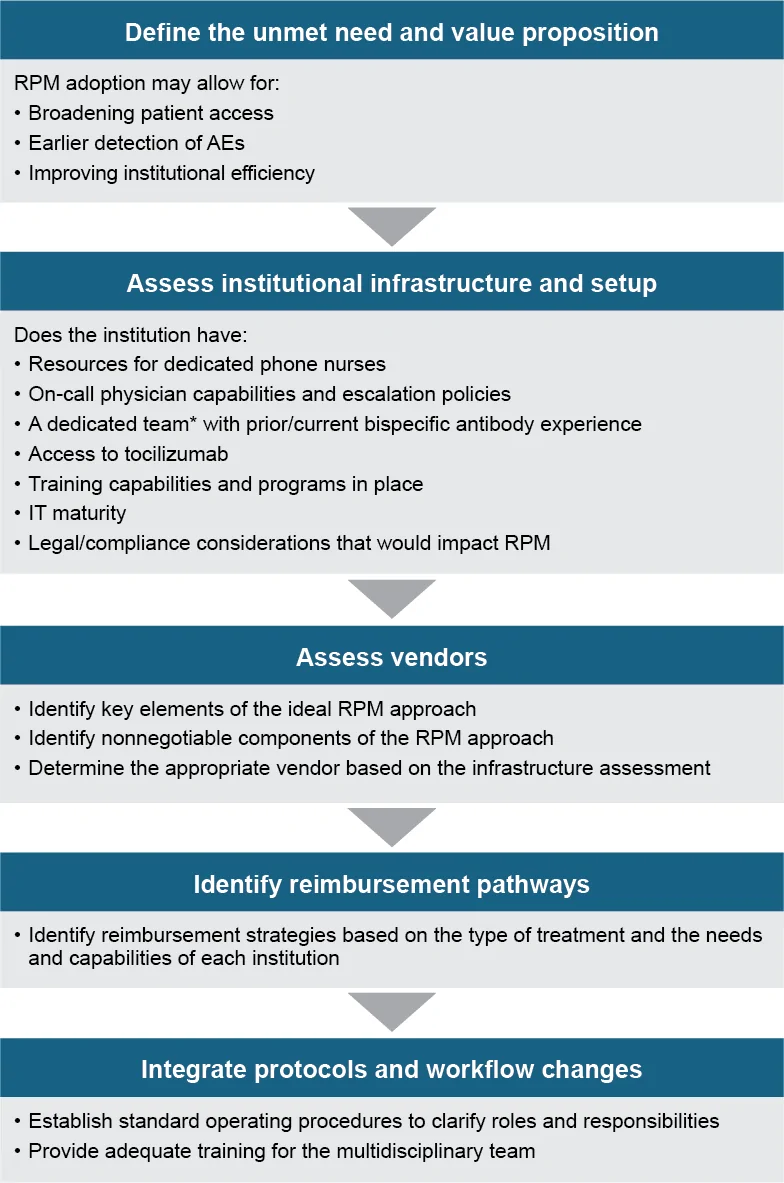
Overview of key steps and considerations for implementing remote patient monitoring (RPM) in outpatient and community settings. *A dedicated team may consist of physicians, nurses (eg, bispecific registered nurses, and advanced practice registered nurses), and pharmacists. AE: adverse event.

## Building the Business Case

Determining the benefits of RPM adoption may vary depending on the type of institution and the specific unmet needs. First, RPM may allow for broadening of patient access to bispecific antibody therapy. In the case of academic institutions, improving hospital bed capacity and expanding treatment throughput with the use of RPM may allow physicians to focus on patients who need more acute care. For community practices, RPM may provide a mechanism to safely retain patients rather than referring them to academic institutions or centers of excellence, enabling these institutions to grow and extend their reach without being limited by traditional resource constraints. Of note, RPM may allow for earlier detection of AEs [36] and better management of care in the outpatient and home settings that would positively impact patients’ quality of life. Moreover, the implementation of RPM may improve institutional efficiency. As novel immunotherapies become more widely used in clinical practice, particularly as approvals are achieved in earlier lines of therapy, RPM will become increasingly more important for managing institutional workload and capacity considerations. With proper implementation and training, RPM has the potential to reduce the overall workload (eg, call volume) for institutions over time.

The successful implementation of RPM depends on the selection of the right technology to meet institutional and patient needs, as well as comprehensive training, institutional buy-in, and stakeholder engagement [34]. RPM may be seen as a new way of care delivery, yet when supported by adequate training and protocols, including patient and caregiver education on the signs and symptoms of potential complications (eg, CRS and ICANS) [21,42], RPM can advance the safe and effective administration of bispecific antibodies in the outpatient setting [31,36-38]. Ensuring the buy-in of all stakeholder groups (including executive leadership and management teams, health care providers [physicians and advanced practice providers, nurses, emergency room physicians, and pharmacists], and administrators) will help ensure successful implementation. Each practice should identify an RPM “champion” who can convey the benefit of RPM to stakeholders and guide the practice through the implementation process. In addition, the caregiver burden must also be considered as they may need to take time off work to care for the patient. For patients receiving bispecific antibodies, it is important for practices to establish standard operating procedures with specific criteria for outpatient SUD dictated by distance from the practice, caregiver availability, comorbidities, and disease characteristics [43] (Table 1).

**Table 1.** Recommended patient selection criteria for remote patient monitoring in outpatient and community settings [21,44].

Domain	Criteria
Disease burden	ECOG^a^ performance status of 0-2 Low tumor burden
Comorbidities	Well-controlled comorbidities Good mental status
Prior CRS^b^ or ICANS^c^	No history of severe CRS or ICANS with prior dosing
Logistical considerations	Close proximity to hospital (eg, within 60 min) Dedicated caregiver support up to 48 h after the first target dose

a ECOG: Eastern Cooperative Oncology Group.

b CRS: cytokine release syndrome.

c ICANS: immune effector cell–associated neurotoxicity syndrome.

While implementing an RPM program can be time-consuming depending on the specific type of RPM and the infrastructure already in place, in the long term, the use of an RPM program will ultimately lead to a more streamlined process and better patient care delivery. Given that RPM models will differ between clinical practices, having a clear understanding of the institution’s individual needs and working with an RPM vendor to tailor an approach specific to the practice can streamline the process and ensure that an effective solution is implemented. Securing institutional alignment is central as organizational support drives smoother implementation and long-term success of RPM.

Stakeholders also need to identify key performance indicators that will measure the success of the RPM program upon implementation, including measures of clinical, operational, and financial success. Clinical success can be measured via patient throughput and improvements in patient outcomes, such as lower-grade CRS events or fewer hospital readmissions. For example, Atrium Health Levine Cancer Institute conducted a study that implemented the use of RPM and prophylactic dexamethasone after each outpatient SUD, which limited the severity of CRS events compared with standard-of-care inpatient monitoring (41% grade 1 events, 19% grade 2 events, and no grade 3 or 4 events with outpatient SUD vs 33% grade 1 events, 12.5% grade 2 events, and 8% grade 3 or 4 events with inpatient SUD) and reduced the incidence of ICANS (6% vs 17%, respectively) [39]. The rate of recurrent CRS with subsequent SUD was also lower with outpatient SUD vs inpatient monitoring (32% vs 46%, respectively). In addition, a reduction in the total days of readmission (42 vs 105 days, respectively) and total inpatient days per patient (1.1 vs 7.8 days, respectively) was observed with outpatient SUD vs standard inpatient SUD.

Operational success can be defined when the RPM system is seamlessly integrated into the daily operations of the practice and those involved can shift their focus to other priorities, indicating that the system is functioning well without constant attention. Improvements in operational workflow result from the nurses or care teams spending less time answering unnecessary calls (allowing them to focus on other responsibilities) as well as from the reduced need for inpatient visits. At Atrium Health, the fielding and triaging of calls by the Hospital at Home team has helped alleviate the burden on providers in the clinic or hospital wards.

Finally, financial success is linked to the clinical and operational metrics (eg, reducing hospital stays and readmissions can lead to cost savings, which are quantifiable financial benefits). Compelling data from Atrium Health Levine Cancer Institute showed that fewer patients who received outpatient SUD with RPM and prophylactic dexamethasone required tocilizumab than those who received inpatient SUD based on the standard-of-care protocol (13% vs 42% of patients, respectively), resulting in reduced estimated total tocilizumab-related per-patient costs (US $868 vs US $2909, respectively) [39]. Overall, the mean estimated cost per patient was US $6444 with outpatient SUD vs US $29,911 with standard inpatient SUD. The use of ePROs at the Highlands Oncology Group to monitor infections in patients with hematologic malignancies has also demonstrated substantial cost savings, with reductions in infection-related hospitalizations and emergency department visits yielding an estimated US $977,695 annual savings per 1000 patients [41]. It is important to note that these results are based on our own single-center experiences; more cost-effectiveness data will emerge as more institutions adopt RPM to manage outpatient administration of bispecific antibodies.

## Infrastructure Assessment and Device Considerations

The existing infrastructure and available resources within a clinical practice should be evaluated prior to RPM decision-making as these elements will inform the appropriate RPM solution. Existing infrastructure may include a dedicated phone nurse, an established electronic medical record (EMR) system (eg, Encore, Flatiron, or Epic), and overall IT maturity, as well as the ability to admit patients in the event of an AE. For example, Highlands Oncology uses an RPM system that functions as a hybrid solution approach, where the Highlands Oncology advanced practice registered nurse system standard operating procedures assist with patient monitoring. This model has decreased the risk of infection-related hospitalization rates by 52% and the risk of infection-related emergency department visits by 33% over 2 years for patients with hematologic cancers [41], and the first exclusive RPM patient was enrolled in July 2026. Moreover, the RPM system needs to be flexible so that it can be adjusted based on the institution’s individual needs. Resources must also be available to provide answers to specific questions and implement algorithms for care (eg, “if patient says x, you do y”). Typically, RPM vendors will offer this type of support (tailored to meet the particular needs of the institution) to help reduce burden on the champion and overall care team [34].

The infrastructure at Atrium Health Levine Cancer Institute exemplifies collaboration between the physicians and the Hospital at Home nurses. This allows for an open line of communication and establishment of a proactive escalation plan that initiates physician involvement when necessary, thereby alleviating the burden of unnecessary calls, and allows for timely intervention to prevent hospitalization. In a case example, the on-call physician received a call from a Hospital at Home nurse about a patient who received SUD at Atrium Health Levine Cancer Institute followed by prophylactic dexamethasone and later experienced a fever. The patient took acetaminophen for the fever, which did not resolve within 1 hour, so the patient was administered an additional dose of dexamethasone. Even though this lowered the fever, the nurse and on-call physician proactively discussed next steps (tocilizumab administration at the infusion center) in case the patient’s fever spiked again. This case study demonstrates how a model of open communication between the physician and Hospital at Home nurse was able to provide timely treatment guidance; develop a proactive escalation plan; and, ultimately, keep this patient with grade 1 CRS out of the hospital.

The evaluation of potential vendors for RPM should be based on the level of existing infrastructure within the institution. An initial assessment of available RPM solutions that considers the practice setup of the institution should be conducted. An assessment of other institutions with established RPM programs should also be conducted. One important consideration is whether the institution has the capacity or resources for dedicated phone nurses or if that component needs to be outsourced to an agency such as Hospital at Home or even the RPM vendor if such services are offered.

There are several factors that can facilitate implementation regardless of the RPM system or device. For example, enthusiasm and an openness to change, logistical maturity to handle patient escalation, and financial maturity to sustain the program will support the initial implementation and contribute to long-term success. Most importantly, all stakeholders need to be appropriately trained on the use of new therapies, including management of common AEs and appropriate triaging and escalation procedures, and education and training can be a time-consuming aspect of integration. Implementation of RPM can be straightforward with adequate training on how to recognize AEs (eg, CRS or ICANS) over the phone, as well as how to administer guidance, advice, and care for patients. On the basis of clinical experience from the Atrium Health Levine Cancer Institute and the Highlands Oncology Group, we recommend that institutions have the following infrastructure in place for the successful adoption of RPM for bispecific antibodies: (1) established hematology and oncology floors with clinicians who are familiar with and comfortable with caring for patients with cancer; (2) a pharmacist who is aware of tocilizumab, a pharmacy that stocks it, and a champion who understands the protocols and how to use tocilizumab; and (3) adequate training for all team members (eg, emergency room and critical care staff, infusion nurses, pharmacists, and hospice nocturnists). In the case of community setting triage, it is recommended to have a “buddy system” with another clinician at a more experienced site. It should be noted that all these needs can be adapted to RPM of patients receiving other types of drugs with different safety profiles and monitoring requirements (eg, CAR T-cell therapy).

Technological systems should be adjustable to various settings and patient demographics. While vendors often provide technological support (eg, implementation managers), some level of IT maturity is required for implementation (eg, an established EMR system). However, it is a misconception that implementation of RPM can only be successful with full EMR integration. In the Current Health model at Atrium Health Levine Cancer Institute, Hospital at Home nurses have a separate dashboard for monitoring vital signs. If there is a change in vital signs, Hospital at Home nurses will document the vital sign change (eg, fever) in the Epic EMR system at Atrium Health, where it is then visible to the physicians. Thus, this monitoring approach can be successful either with or without full integration into the hospital’s EMR system. It is also important to remember that limited resource barriers can be overcome. Having well-trained nursing staff available to answer patient calls and triage patients with serious AEs is critical. However, if capacity does not allow for internal phone nurses, outsourcing provides an option to eliminate this barrier, such as the Current Health Clinical Command Center. In addition, while pilot studies are recommended, they are not required. Pilot studies can provide learnings that inform a scale-up program; however, to be successful, a pilot study needs to be approached as a priority project that is well planned and executed with intention of full deployment in the future.

## Reimbursement and Financial Challenges and Considerations

In the case of bispecific antibodies, RPM is currently not considered part of chronic care management in the United States, making billing and reimbursement something that needs to be established. While virtual visits with a physician can be billed to insurance, clinicians may not have established billing requirements for RPM, which is the case for the Atrium Health Levine Cancer Institute. In some programs, however, the cost savings of monitoring patients in an outpatient setting help offset the costs associated with RPM. Reimbursement considerations differ for Highlands Oncology as insurance payments do not cover the cost of an overnight nurse call and advanced practice registered nurse call. Additional billing options, including telehealth, are also being considered. In the United States, there are 9 reimbursement codes for RPM of physiological parameters in 2026, including a new code that covers RPM device supply and data transmission for 2 to 15 days of a 30-day period (Current Procedural Terminology code 99445) [45]. This code is particularly relevant for patients receiving bispecific antibodies who need to be monitored in the first few days after outpatient SUD.

Reimbursement strategies will depend on the type of treatment as well as the needs and capabilities of each institution. For some institutions, reimbursement for RPM may occur through billing codes as part of service. In the event that RPM is not included as part of the billing code, practices will need to explore other reimbursement mechanisms. In some cases, device provisioning can be considered, allowing the institution to deliver and manage the device. At Atrium Health Levine Cancer Institute, RPM (including monitoring or paramedic visits) does not incur a separate fee; the patient or insurance company is charged only for the virtual visits with the internist from Hospital at Home.

## Contracting and Legal Considerations and Liabilities

Institutional buy-in and commitment can drive urgency and expedite the process of RPM adoption and implementation. Academic medical centers may face more bureaucratic hurdles than community practices. In academic medical centers and larger institutions, contracting can be a significant source of delays due to the extensive processes and guardrails involved, such as clinical implementation, data sharing, and IT requirements. These centers typically have more complex procedures to navigate. For example, integrating RPM devices into a large already established EMR system can introduce additional complexities, requiring collaboration between the institution and the vendor. Despite this, the benefits of the RPM systems and care models outweigh this potential hurdle. Conversely, community and independent practices generally face fewer contractual and logistical hurdles, allowing for a nimbler approach. While contracting can be a time-consuming part of the RPM process, implementation can be rapid once all paperwork has been signed, a process that is generally made smoother with institutional buy-in and input.

The legal liability of RPM is similar to that of traditional care, but the main challenge is adapting to the new paradigm of remote care. Physicians must accept some risk in remote monitoring, balanced by the benefits of outpatient care. Patients are no longer under constant direct supervision as they would be in a hospital, and this shift in thinking can be challenging for physicians who are accustomed to being able to check on patients in person in the event of a concern. Accepting this new model requires trust and confidence in the benefits of outpatient care for both the patients and the team of health care providers. Ultimately, it is up to the physician to determine the right balance between direct involvement and RPM, and this requires a personalized approach for each patient.

## Potential Limitations of RPM

While RPM may offer several advantages for patients and providers, it is not without limitations. Programs will need to consider how to manage device malfunction or failure and ensure that patients have sufficient internet connectivity [34,46]. Some patients may have slower connections and data caps that could limit transmission of data; however, this can be addressed using RPM gateways that connect directly to providers and bypass the patient’s home network [46]. Robust security measures are needed to prevent unauthorized access to patient data and protect patient privacy [46]. Digital literacy gaps may impact the success of RPM, particularly among older adult patients who may find new technologies disorienting [46-48]. Socioeconomic barriers may also limit access to and adoption of RPM [46,47]. Proactive engagement and education are needed to ensure inclusive implementation of RPM for all eligible patients [48].

## Conclusions

The practical recommendations discussed herein give health care providers the tools needed to successfully implement RPM for patients receiving bispecific antibodies in outpatient clinics and nonacademic centers, giving patients with MM broader access to bispecific antibodies and leading to improved patient outcomes. The use of RPM for patients with MM receiving bispecific antibodies has the potential to profoundly improve patient care and the patient experience. Potential benefits include increased access to care, earlier AE detection and better AE management, and lower inpatient hospitalization time. By shifting the way in which health care providers think about patient care, RPM can also improve efficiencies in the health care system by reducing resource burdens (eg, hospital staffing and capacity) and lowering overall health care costs. Community practices often refer their patients to academic centers for the SUD phase [42] and may lose them as a consequence. Decentralizing SUD for bispecific antibodies to community physicians empowers them to remain central in the care of their patients rather than transferring management exclusively to tertiary care centers. This allows for continuity of care, potentially leading to better outcomes, and facilitates trust and comfort between the patient and health care team.

Taken together, RPM implementation is a feasible strategy to optimize outpatient dosing of bispecific antibodies for both academic and community practices, and specific approaches will vary based on the needs and goals of each treatment center. Importantly, RPM protocols and management pathways are broadly applicable to other drugs (eg, CAR T-cell therapy [49]), different types of AEs, and diseases outside the oncology setting.

## Abbreviations

AE: adverse event
CAR: chimeric antigen receptor
CRS: cytokine release syndrome
EMR: electronic medical record
ePRO: electronic patient-reported outcome
ICANS: immune effector cell–associated neurotoxicity syndrome
MM: multiple myeloma
RPM: remote patient monitoring
SUD: step-up dosing
